# Contrast Media–Induced Anaphylaxis Causing a Stress-Related Cardiomyopathy Post Percutaneous Coronary Intervention: Case Report

**DOI:** 10.1177/2324709617712735

**Published:** 2017-06-01

**Authors:** Rajeev Seecheran, Valmiki Seecheran, Sangeeta Persad, Sasha Lalla, Naveen Anand Seecheran

**Affiliations:** 1 The University of the West Indies, Mt. Hope, Trinidad and Tobago; 2 North West Regional Health Authority, Port-of-Spain, Trinidad and Tobago; 3 Advanced Cardiovascular Institute, Port of Spain, Trinidad and Tobago

**Keywords:** anaphylaxis, hypersensitivity, contrast media, epinephrine, stress-related cardiomyopathy

## Abstract

Anaphylaxis is a sudden-onset, severe hypersensitivity reaction that can be potentially fatal. It can often transition to refractory hemodynamic instability, eventually resulting in death. Stress-related cardiomyopathies (SRCs) have multifactorial etiologies, including being linked to excessive catecholamine release in periods of intense stress. This novel case report recounts a SRC caused by contrast-induced anaphylaxis within 1 hour post percutaneous coronary intervention. Both acutely life-threatening conditions may occur simultaneously and are implicated with devastating complications. Further research is required to understand this cardiac-neuroaxis interplay in SRC to identify risk factors and develop management strategies.

## Introduction

Anaphylaxis is a sudden-onset, severe hypersensitivity reaction that can be potentially fatal. It can often transition to refractory hemodynamic instability, eventually resulting in death.^1^ There has been a drastic rise in the use of radiographic contrast media (RCM) for both diagnostic testing and therapeutic interventions. As a result, hypersensitivity reactions are now frequently encountered because of the increased utilization of RCM. These reactions may be classified as immediate (less than 1 hour of administration) and nonimmediate reactions (greater than 1 hour).^2^ With the recent advent of low-osmolality, nonionic contrast media, the incidence of immediate RCM hypersensitivity declined precipitously from 3.8% to 12.7% to 0.7% to 3.1% as compared to high-osmolality, ionic types.^3^ Similarly, the incidence of the severe immediate reactions also decreased from 0.1% to 0.4% to 0.01% to 0.04%.^4^ Presently, the clinical characteristics for the development of anaphylaxis are not well defined; however, cardiovascular involvement often remains a critical feature.

Stress-related cardiomyopathies (SRCs) can occur in several situations, two of which may be applicable in this clinical scenario, namely, myocardial dysfunction (left ventricular dysfunction [LVD]) associated with external catecholamines, for example, epinephrine administered during resuscitation, and new-onset LVD in tenuous, otherwise en extremis patients.^5^ They are also more likely to occur in middle-aged women. ^6^ SRCs have multifactorial etiologies, including being linked to excessive catecholamine release in periods of intense stress. There currently exists a paucity of data, specifically with respect to contrast media–induced anaphylaxis causing a SRC, and we present this as novel case for such.

## Case Report

A 51-year-old South Asian woman with a medical history of hypertension and dyslipidemia presented with Canadian Cardiovascular Society class II angina for the preceding 6 months. She was initiated on optimal medical therapy including low-dose aspirin 81 mg, a beta-blocker, and a high-intensity statin and risk stratified with an exercise stress echocardiogram. The stress test revealed intermediate-risk findings with a Duke Treadmill Score of −3, a preserved left ventricular ejection fraction (LVEF) of 60%, and severe anterolateral hypokinesis. Subsequently, she was scheduled for coronary angiography with informed consent for likely ad hoc percutaneous coronary intervention (PCI). Her physical examination, vital signs, and routine blood investigations were normal. A 12-lead electrocardiogram (EKG) revealed normal sinus rhythm with no evidence of acute ischemia or prior infarction.

**Figure 1.**
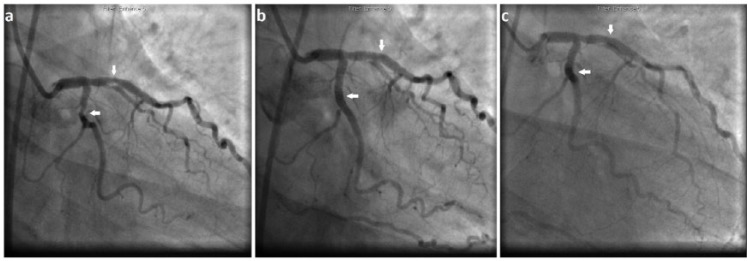
(a) Left coronary artery angiography with solid white arrows indicating the ACC/AHA Type A left anterior descending (LAD) and circumflex (CFx) lesions. (B) Post drug-eluting stent, percutaneous coronary intervention (DES PCI) ×3 with good angiographic result (TIMI 3 antegrade flow with no evidence of dissection or perforation). Solid white arrows indicate the improved appearance with good stent expansion and apposition. (c) Emergent coronary angiography postresuscitation with white solid white arrows revealing widely patent stents and TIMI 3 antegrade flow in both LAD and CFx.

Coronary angiography revealed proximal left anterior descending coronary artery (LAD) and mid circumflex coronary artery (CFx) ACC/AHA type A stenoses (see Figure 1a). The patient’s calculated SYNTAX II score was 8. Transfemoral PCI was performed and a total of 3 SYNERGY (Boston Scientific, Marlborough, MA) drug-eluting stents were successfully implanted into the LAD (1 stent) and CFx (2 stents) with a good angiographic result and no apparent complications (see Figure 1b). A total of 200 cc of Ultravist 300 (Bayer HealthCare LLC, Whippany, NJ) was used.

**Figure 2.**
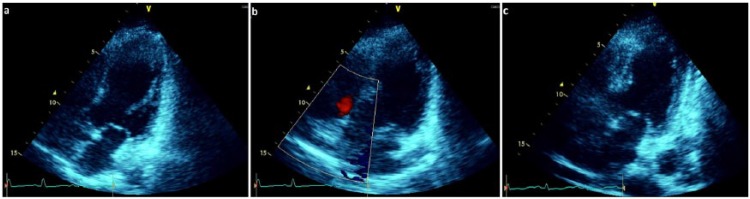
(a) Transthoracic echocardiogram (TTE) parasternal long-axis view revealing severe left ventricular function of 20% to 25% with apical ballooning and spontaneous “echo-smoke.” (b) TTE apical 2-chamber view showing apical ballooning. (c) TTE apical 2-chamber off-axis view showing apical ballooning and dyskinesis.

Approximately 1 hour postprocedure, the patient became hemodynamically unstable with severe hypotension, hypoxia, and tachycardia. Her tenuous vital signs reflected a blood pressure of 80s/50s mm Hg with a heart rate of 140s beats per minute and saturating 84% on room air. She had diffuse urticaria on her chest wall and upper extremities and reported severe dyspnea with intractable nausea and vomiting. She then became unresponsive and deteriorated into cardiopulmonary arrest with asystole. The likely differential diagnoses at this juncture included contrast media anaphylaxis, severe coronary vasospasm with right ventricular dysfunction, and acute stent thrombosis (AST) with cardiogenic shock. She had return of spontaneous circulation (ROSC) after ~10 minutes of the advanced cardiac life support (ACLS) protocol during which she received 2 mg intravenous epinephrine, 200 mg intravenous hydrocortisone, 100 mg intravenous ranitidine, and 10 mg intravenous chlorphenamine for anaphylaxis, along with other standard-of-care ACLS therapies. Simultaneously with the ongoing resuscitative efforts and anaphylaxis treatment, an emergent angiogram via the preexisting femoral access site sheath revealed widely patent stents in both arteries, each with TIMI 3 antegrade flow (see Figure 1c), not indicative of vasospasm or AST. A bedside transthoracic echocardiogram revealed a severely depressed LVEF of 20% to 25% with apical ballooning similar to that of a Takotsubo cardiomyopathy, without evidence of cardiac tamponade or right ventricular dysfunction (see Figure 2a-c). An arterial blood gas indicated an acidemia consistent with type 1 respiratory failure.

Based on the clinical scenario, the patient’s tentative diagnosis was contrast media–induced anaphylaxis and cardiogenic shock due to an SRC. She was subsequently transferred to the cardiac care unit for further management, which comprised emergent intubation by the intensive care team, followed by mechanical ventilation and dual inotropic infusion with moderate-dose epinephrine and dopamine. Her cardiac biomarkers were minimally elevated (peak serum levels of creatine phosphokinase-MB fraction and troponin T were ~10 ng/mL [normal range = 1-4 ng/mL] and ~2 ng/mL [normal range = 0-0.4 ng/mL]; both at day 2), respectively, and did not reflect a periprocedural myocardial infarction. A post–cardiac arrest EKG indicated a sinus tachycardia with nonspecific changes. On day 3 of hospitalization, a 2D-transthoracic echocardiogram showed near-normalization of LVEF to 50% to 55% without regional wall motion abnormalities. The patient gradually became more hemodynamically stable over the ensuing hospitalization, requiring less vasopressors and ultimately did not require an intra-aortic balloon pump. She was safely discharged on guideline-recommended, optimal medical therapy (OMT) comprising dual antiplatelet therapy with clopidogrel, novel oral anticoagulant rivaroxaban, beta-blocker, angiotensin-converting enzyme inhibitor, aldosterone receptor antagonist with high-intensity statin, as well as a tapered course of oral steroid therapy.

## Discussion

SRCs are now emerging as a significant contributor of myocardial dysfunction within the perioperative window, and its prevalence needs to be established in large-scale, prospective cohort studies. This novel case report presents an anaphylactic response to contrast media resulting in an SRC. The etiology of the SRC was likely multifactorial, attributed to both LVD associated with the critical physiological state from the fulminant anaphylaxis and subsequent exogenous catecholamine administration (a total of 2 mg intravenous epinephrine) during resuscitation. Resuscitation upregulates the sympathetic nervous system and increases serum catecholamine concentrations, which could potentiate cardiac dysfunction.^7^ Several basic science studies suggest that epinephrine mediates this dysfunction through its β_1_-adrenergic actions.^7^

In anaphylaxis, systemic vasodilatation, reduced venous return, and volume loss due to increased vascular permeability lead to a diminished cardiac output, which contribute to coronary hypoperfusion and subsequent myocardial damage. Coronary vasospasm has been proposed as a putative central mechanism, while additionally being implicated in SRCs^8^; however, this phenomenon was not visualized on emergent postresuscitation angiography. It has also been postulated that the systemic catecholamine increase with excessive activation of cardiac catecholamine receptors in the left ventricle plays a major role in the pathophysiology of SRC.^9^ The catecholaminergic storm, either exogenous or endogenous, may cause direct myocardial toxicity. Microvascular dysfunction resulting in myocardial stunning may also be involved.^10^ Overall, this double-hit phenomenon resulted in a neurohormonal milieu and cascade amplification of endogenous epinephrine and norepinephrine levels, which was potentially exacerbated by the administration of the intravenous epinephrine boluses followed by a moderate-dose infusion for several days.

In 2005, a study of patients admitted to the medical intensive care unit discovered that LV apical ballooning occurred in 28% of cases using echocardiography^11^; however, coronary angiography was not performed and, thus, the diagnosis of SRC could not be fully ascertained. This patient had a protracted recovery over a 7-day hospitalization. The mainstay of therapy included intravenous steroids, weaning vasopressors, and ventilator settings as she improved clinically and also with respect to serial echocardiography. Treatment of SRC is fundamentally based on conventional therapies, for example, inotropic support with mechanical ventilation and judicious fluid management. Patients’ outcomes are usually benign if they survive the initial aspect of the hospitalization, as life-threatening issues can arise during this period. Cardiac dysfunction may be initially severe; however, it is transient and generally responds in a favorable manner with normalization of left ventricular function.^12^ Heart failure, cardiogenic shock, and death occur in 3.8%, 6.5%, and 3.2% of patients, respectively.^13^ A myriad of other rare complications include dysrhythmia, cerebrovascular accident, left ventricular outflow tract obstruction, left ventricular thrombus formation, and free wall rupture.^14^

Currently, there are no established guidelines on premedication for RCM-induced anaphylaxis^15^; however, corticosteroid prophylaxis remains the standard of care in the United States.^16^ Both oral and parenteral steroids are often administered in combination with H1-antihistamine and H2-histamine receptor blockers; however, these regimens cannot ensure complete prevention of RCM-induced anaphylaxis. The principle of postanaphylaxis management is to avoid the causative agents. With respect to our patient, the anaphylaxis was a de novo, idiosyncratic event, and thus, we did not premedicate her for angiography with ad hoc PCI. Some studies suggest that lower doses of epinephrine or longer dosing intervals may be prudent after ROSC^17^; however, pending more conclusive data or a formal change in ACLS protocols, we suggest giving epinephrine in accordance with the existing guidelines as we did with our patient.

## Conclusion

In summary, we report a novel case of SRC caused by contrast-induced anaphylaxis within 1 hour post-PCI. Both acutely life-threatening conditions may occur simultaneously and are implicated with devastating complications. The heart team should be cognizant that this left ventricular dysfunction is often severe, fully reversible, and should be managed with supportive therapies, preferably in an intensive care setting. Surveillance echocardiography during hospitalization may play a pivotal role in guiding treatment as demonstrated in this case. Further research is required to understand this cardiac-neuroaxis interplay in SRC and to identify risk factors and develop management strategies.
